# Differences in Postural Balance, Pain Sensitivity and Depression between Individuals with Acute and Chronic Back Pain

**DOI:** 10.3390/jcm11102700

**Published:** 2022-05-10

**Authors:** José Antonio Mingorance, Pedro Montoya, José García Vivas Miranda, Inmaculada Riquelme

**Affiliations:** 1 Research Institute on Health Sciences (IUNICS-IdISBa), University of the Balearic Islands, 07122 Palma de Mallorca, Spain; pedro.montoya@uib.es (P.M.); inma.riquelme@uib.es (I.R.); 2 Department of Nursing and Physiotherapy, University of the Balearic Islands, 07122 Palma de Mallorca, Spain; 3 Laboratory of Byosystems, Institute of Physics, Federal University of Bahia, Salvador 40170-115, Brazil; vivasm@gmail.com

**Keywords:** proprioception, postural balance, acute pain, chronic pain, depression

## Abstract

To compare differences in postural balance, pain and depression in patients with chronic and acute low back pain, twenty patients with chronic and twenty patients with acute low back pain from the Edward Francis Small Hospital (Banjul, Gambia), as well as 20 age-matched healthy controls participated in the study. A modified Romberg test was used to assess postural balance during one minute with closed eyes. Body sway in the anteroposterior and mediolateral axes was video-recorded during test performance and further analyzed with an open source software for movement analyses (CvMob). Pain sensitivity was assessed by means of pressure pain thresholds and depression by a self-report questionnaire (PHQ-9). As results, patients with chronic low back pain displayed higher body sway in the anteroposterior and mediolateral axes, as well as faster body sway than patients with acute low back pain and healthy controls. Nevertheless, group differences disappeared when depression was introduced as a covariate, indicating a major role of depression in postural balance deficits of patients with pain disorders. As conclusions, the assessment of postural balance and depression should be implemented in the clinical routine for the design of tailored interventions in pain conditions.

## 1. Introduction

Postural balance is compromised in patients with chronic pain, such as complex regional pain syndrome [1], fibromyalgia [2], neck [3,4] and low back pain [5,6]. One possible explanation is that these deficits could be due to central processes involved in pain experience. Indeed, it has been shown that altered postural balance in patients with chronic low back pain could be associated with changes in motor cortex organization [7]. Accordingly, several studies have reported that an alteration in motor control may have causative impact on the emergence and maintenance of chronic pain [8,9,10,11,12,13].

The transition from acute to chronic pain is determined by many pain features and individual characteristics [14]. In this sense, depression has been considered one of the main risk factors for pain chronification [15,16,17]. Thus, it has been reported that depression can predict the persistence of pain in muscle-skeletal injuries [18], contributes to the transition towards chronic pain [19,20,21], and is associated with postural instability in neurological disorders such as stroke [14] and Parkinson disease [20], as well as in the elderly [22].

The impairment of postural balance in chronic pain conditions is clinically relevant due to its association with the risk of falls and functional restrictions in daily life [21]. Determining whether postural dysfunction is present in acute and chronic pain, as well as examining its relationship with the presence of depression, could contribute to improve the treatment and diagnosis of pain in several pain conditions [3]. The present study aimed at exploring potential deficits in postural balance associated to pain duration, pain sensitivity and depression in individuals suffering from acute or chronic low back pain. For this purpose, pressure pain sensitivity at a painful and a non-painful body location, self-reports of depression and several parameters of static body sway in patients with acute or chronic low back pain were compared to healthy individuals. We hypothesized that postural balance would be impaired in individuals with chronic pain as compared to acute pain patients and healthy controls, and that deficits in postural balance would be modulated by depression.

## 2. Materials and Methods

### 2.1. Participants

Individuals with chronic and acute low back pain were identified by medical doctors at the Edward Francis Hospital (Banjul, Gambia) in summer of 2016. Chronic pain was defined as pain lasting more than 3 months. Inclusion criteria were: [1] age between 25 and 50 years, and [2] diagnosis of low back pain at the acute or chronic phase. The selected patients were informed of the aim and methods of the study and invited to participate by signing the informed consent. The protocol was approved by the Research and Ethics Committee of The Republic of the Gambia. Participants were excluded from the study if they had not signed the informed consent or if a diagnosis of neurological disorders were included in the hospital medical report.

The mean low back pain point prevalence in Africa is 32% [23]. A sample size calculation was performed taking into account the Banjul’s population of 33,000 inhabitants, and by using the GRANMO sample size calculator (GRANMO: http://www.imim.es/) with a power of 0.9 and alpha of 0.05. According to these parameters, it was estimated that a sample of 20 participants per group would be required to detect significant differences. Forty people with low back pain accepted to participate and were included in the study: 20 patients with acute low back pain [15 females, mean age = 37.9 (1.32)], and 20 patients with chronic low back pain [12 females, mean age = 40.8 (1.44)]. Twenty age-matched healthy individuals with no pain [15 females, mean age = 40.8 (1.63)] were also recruited and included in the study. Group comparisons for age, body mass index, height, weight and pain duration are displayed in Table 1.

### 2.2. Assessments

Postural balance, pressure pain sensitivity and level of depression were assessed in all participants in one session at the Edward Francis Hospital.

Pain sensitivity was assessed by using a standard algometer and was defined as the necessary pressure (expressed in Newtons) to cause a painful sensation. Algometry was applied at two bilateral body locations following a pseudorandom sequence: great trochanters (defined by low back pain patients was a painful body location) and epicondyles (non-painful body location). Algometry has been found to be non-invasive, efficient and reliable [24] in the exploration of pathophysiological mechanisms involved in muscle pain syndromes [25] and is considered as a neurophysiological marker of central somatosensory processing [26].

Depression was assessed by the PHQ-9 scale of The Patient Health Questionnaire. This self-report questionnaire is considered as a good screening tool for depression in primary care [24].

Postural balance was assessed by asking participants to perform the modified Romberg test for one minute and with their eyes closed. This task has proven to be an objective measure of balance in an upright position [27]. The task performance was recorded on video with a standard webcam (©Logitech, Lausanne, Switzerland) at a rate of 30 frames per second and located two meters above ground level. For recording purposes, participants used a headband (located at the level of the parietal lobe) that contains two marks separated 5 cm from each other. Participants were asked to remain in an orthostatic position with their feet apart (at shoulder width) and with arms extended along the body [17]. The balancing of the body in the mediolateral and anteroposterior axes was processed through the use of an open source software (CvMob) developed for computer vision purposes [27]. The standard deviation of body sway in each plane (mediolateral and anteroposterior), as well as speed of body sway (cm/sec) were obtained as balance parameters. It has been shown that the measurement and analysis of body sway through this procedure are reliable and produce results similar to those provided by posturography [27].

### 2.3. Statistical Analyses

The assumption of normality in all variables was previously assessed with the Kolmogorov-Smirnov test. Analyses of variance (ANOVAs) were used to test group differences (between-subject factor GROUP: chronic pain vs. acute pain vs. healthy controls) in postural balance, pressure pain sensitivity and level of depression. An additional within-subjects factor BODY LOCATION (epicondyle vs. greater trochanter) was used to analyze pressure pain sensitivity. Finally, the within-subjects factor AXIS (anteroposterior vs. mediolateral) was also used to analyze balance parameters (standard deviations and speed of body sway). Greenhouse–Geisser corrections were applied for the violation of sphericity assumptions in the ANOVAs. Bonferroni corrections were applied for post-hoc comparisons. Pearson correlations were used to explore the associations of body sway parameters with depression and pressure pain. Statistical analyses were performed using the SPSS software. A *p*-value of 0.05 was used for statistical significance.

## 3. Results

There were no significant group differences in age, gender, height, weight or body mass index (*p* > 0.29). As expected, significant differences in pain duration (F(2,57) = 125.65, *p* < 0.001) revealed that patients with chronic pain had longer pain duration than patients with acute pain or healthy controls (all *p* < 0.001).

**Figure 1:**
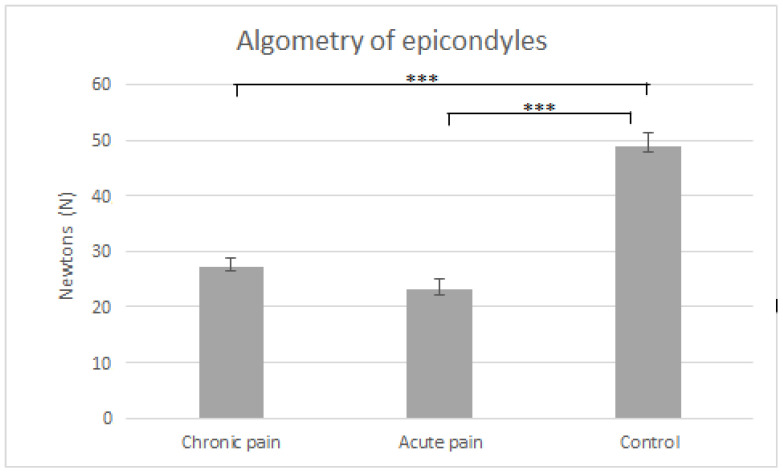
Means and typical errors of pressure pain thresholds (in Newtons) averaged for epicondyles in the three groups (chronic pain vs. acute pain vs. control). *** *p* < 0.001.

**Figure 2:**
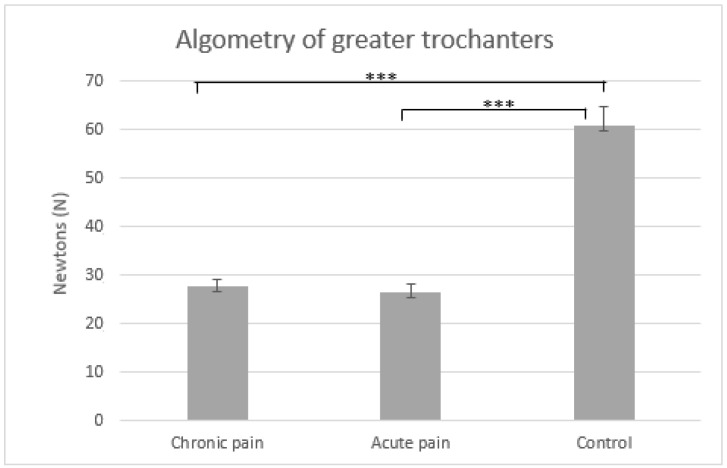
Means and typical errors of pressure pain thresholds (in Newtons) averaged for greater trochanters in the three groups (chronic pain vs. acute pain vs. control). *** *p* < 0.001.

Figure 1 and Figure 2 display means and typical errors of pressure pain measures for the three groups in epicondyles and greater trochanters. Significant effects of GROUP (in epicondyles F(2,57) = 50.66, *p* < 0.001 and in greater trochanters F(2,57) = 50.66, *p* < 0.001), BODY LOCATION (in epicondyles F(1,57) = 12.07, *p* < *0*.001 and in greater trochanters F(2,57) = 13.56, *p* < 0.001) and GROUP × BODY LOCATION were found (in epicondyles F(2,57) = 6.76, *p* = 0.002 and in greater trochanters F(2,57) = 7.66, *p* = 0.003). Post-hoc comparisons indicated that pain thresholds in epicondyles and greater trochanters were lower in patients with chronic and acute low back pain than in healthy controls for both body locations (*p* < 0.001), and that there were no significant differences between patients with chronic and acute low back pain (*p* > 0.40). In addition, post-hoc comparisons revealed that pain sensitivity at the greater trochanter was lower than at the epicondyle (both *p* < 0.001) in healthy controls, while there were no differences between both body locations in patients with chronic or acute pain for both body locations (*p* > 0.19).

**Figure 3:**
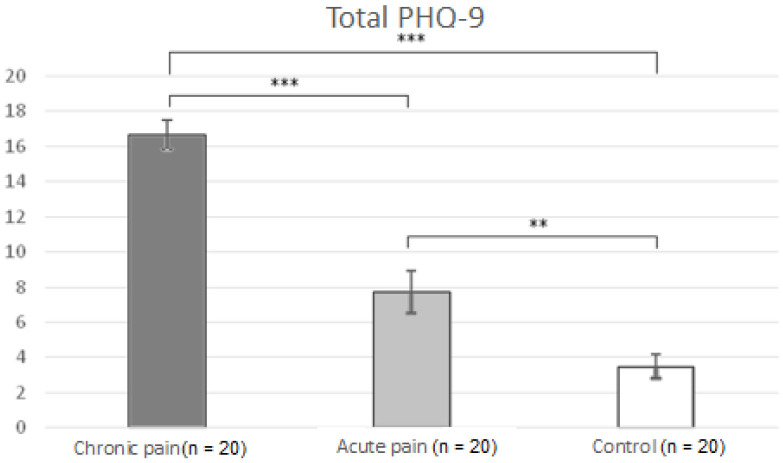
Means and typical errors of depression scores in the three groups (chronic pain vs. acute pain vs. control). ** *p* < 0.01, *** *p* < 0.001.

Figure 3 displays mean depression scores in the three groups. Significant effects due to GROUP (F(2,57) = 51.54 (*p* < 0.001) were found. Post-hoc comparisons indicated that patients with chronic low back pain displayed higher scores compared to patients with acute pain (*p* < 0.001) and healthy controls (*p* < 0.001), and that patients with acute pain reported higher depression scores than healthy controls (*p* < 0.007).

**Figure 4:**
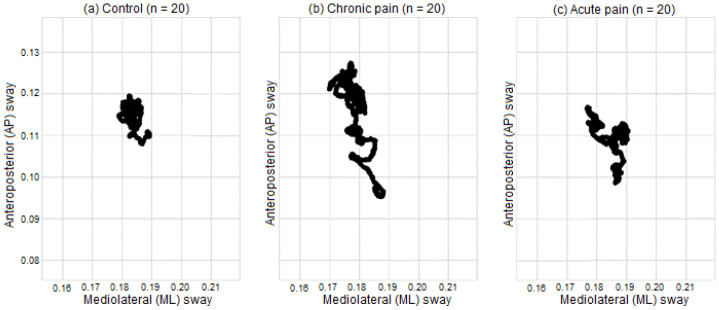
Pattern of body sway in typical individuals from each group. (**a**) Healthy controls, (**b**) patient with chronic low back pain, (**c**) patient with acute low back pain.

Figure 4 shows the pattern of body sway in one typical individual from each group. It can be observed that patients with chronic and acute low back pain display higher variability of body sway as compared to healthy controls. A significant effect due to GROUP was found (F(2,57) = 15.48, *p* < 0.001), which indicates that body displacements in the anteroposterior and mid-lateral axes displayed greater variability in patients with chronic low back pain than in patients with acute pain (*p* < 0.001) and healthy controls (*p* = 0.002), whereas no significant differences were found between patients with acute pain and healthy controls (*p* = 0.19). No significant differences were found due to AXIS (F(1,57) = 2.98, *p* = 0.09) or to GROUP × AXIS (F(2,57) = 1.91, *p* = 0.16).

The ANOVA on speed of body sway yielded a significant effect due to GROUP (F(2,57) = 9.71, *p* < 0.001), showing that body sway was faster in patients with chronic low back pain than in patients with acute pain (*p* = 0.001) and healthy controls (*p* = 0.002), and that there were no significant differences between patients with acute pain and healthy controls (*p* = 0.95).

Finally, significant positive correlations were found between depression scores and body sway parameters (standard deviation of anteroposterior and mediolateral sway, as well as speed of body sway) (all *r* > 0.30, all *p* < 0.02), indicating that impaired body sway parameters were associated with higher depression. No significant correlations were found between body sway parameters and pain sensitivity measures. Considering these results, additional analyses of covariance (ANCOVAs) were performed on body sway parameters controlling for the effects of depression. There were significant effects due to GROUP in body sway through the mid-lateral axis (*p* = 0.005), but not in body sway through the anteroposterior axis or in speed of body sway.

## 4. Discussion

The aim of the present study was to analyze differences on postural balance, pain sensitivity and depression due to chronic and acute low back pain. In particular, we measured the variability of the body sway in the anteroposterior and mediolateral axes, as well as body sway velocity, pain sensitivity at the greater trochanter (painful body location in low back pain) and epicondyle (a usually non-painful body location), and self-reports of depression. Our data revealed that patients with chronic low back pain had poorer postural balance (greater variability and faster body sway velocity) and enhanced depression than patients with acute low back pain, and that the latter were similar to healthy controls in postural balance. In addition, we observed that both groups of patients with acute and chronic low back pain had greater sensitivity to pain than healthy controls. Finally, we found that depression, but not pain sensitivity, accounted for the deficits in postural balance.

These findings are in agreement with previous literature that shows that patients with chronic pain have deficits in postural balance [3,4,9] and, therefore, a greater risk of falls than healthy controls [21]. Indeed, alterations on postural control such as larger sway areas, greater center of gravity displacement or increased EMG activity have been previously described [4,5,6,7,8,9,10,11,12] in patients with chronic low back pain, suggesting some process of reorganization of the central nervous system in response to the chronification of pain [25]. Thus, the fact that only patients with chronic, but not with acute low back pain displayed deficits in postural balance seems to be in agreement with this interpretation. Furthermore, the significant relationship between postural balance deficits and enhanced depression scores seems to provide additional evidence of some type of neural adaptation induced by a central sensitization that goes beyond enhanced pain sensitivity [9].

In the present study, we further observed that both groups of patients with low back pain had enhanced pain sensitivity at painful and non-painful body locations as compared to healthy controls. Chronic low back pain has been associated with widespread changes in somatosensory sensitivity (included enhanced pain sensitivity at locations distinct from painful body regions), pointing to significant brain plasticity and central sensitization [28]. The fact that patients with acute low back pain also showed an enhancement of pain sensitivity at painful and non-painful body locations suggested that maintaining pain for a few days (as in acute low back pain) may also lead to relevant changes in brain processing of pain.

Depression, and not pain sensitivity, was the main factor that influenced postural balance in the present study. Moreover, we found higher levels of depression in patients with chronic pain compared to those with acute pain and healthy controls. These findings are partially in agreement with previous studies that show that altered postural control in patients with chronic pain could be strongly associated with pain-related symptoms [2,8]. Depression and pain are very comorbid and higher levels of depression have been associated with increased clinical sensitivity to pain and functional disability [19]. Furthermore, previous studies have shown that depression is related to deficits in visual and proprioceptive integration [22], can affect the performance of the sensorimotor task and the effectiveness of fall prevention [20], and is associated with a deteriorated balance in neurological conditions such as stroke [14] or Parkinson disease [20]. Therefore, the strong association observed in our study between balance parameters and depression further confirms the role of mood as a key component for postural control and supports the evaluation of depression in clinical routine to adapt the physical intervention in patients with musculoskeletal pain. The link between depression and chronic pain works in both directions [29] It has been shown that emotional disorders, such as depression, are common comorbid conditions that exacerbate the severity and persistence of chronic pain [30]. Pain and depression have been shown to be highly intertwined and can exacerbate physical and psychological symptoms [29,30] Moreover, it has been observed that the serotonergic and norepinephrine system plays a very important role in this comorbidity and that the brain structures that encode pain are also involved in mood, so the use of serotonergic antidepressants and norepinephrine may be useful to mitigate the pain [30,31]. Although more research is needed to analyze the neuroplastic mechanisms that link pain chronification and balance disorders, our findings suggested that the evaluation of postural balance, along with pain sensitivity and depression, could provide powerful indicators for the transition of acute pain to chronic pain.

Our findings should be analyzed taking into account some methodological limitations. One of the major limitation of the present study is that postural balance was measured through the video recording of head movements. Although this method has been previously validated, other direct and indirect measurements of postural control such as posturography (usually recorded by force platforms), electromyography (EMG) or even electroencephalography (EEG) recordings from motor cortices would be helpful to confirm these findings. A second important limitation was that the effects of chronic and acute pain on postural balance were based on a cross-sectional study with a relatively small sample of patients. A larger sample of patients with all possible pain duration intervals could have contributed to a better understanding of the changes that occur in the postural balance associated with the transition from acute to chronic pain. Although in our study there were no sex differences in sensitivity to pain and depression, other studies have shown changes in these variables with respect to gender [32,33,34]. The small sample size may also explain this fact. Another major shortcoming was the lack of data on the current pain perception, apart from pain sensitivity and depression measures. No other psychosocial factors related to pain were collected, such as catastrophizing, pain vigilance, perceived health quality, the impact of pain on social life, or functional disability caused by pain. Finally, the fact that the existence of neurological disorders was only obtained from the clinical records of the patients and was not directly confirmed by the experimenters, could be also considered a relevant shortcoming of the study.

## 5. Conclusions

The findings of this study provided empirical evidence that patients with chronic low back pain had worse postural balance and enhanced depression than patients with acute low back pain. In contrast, patients with acute and chronic low back pain showed a similar enhancement in pain sensitivity. Finally, we found that depression, but not enhanced pain sensitivity, accounted for the deficits in postural balance. All these findings revealed the different relevance of postural balance, pain sensitivity and depression in the transition from acute to chronic pain conditions. Further investigation of the neurophysiological mechanisms involved in the association between these variables could help to better understand the chronification of pain and improve clinical assessment and intervention of chronic and acute low back pain.

## Figures and Tables

**Table 1:** Displays the sociodemografic data of the three groups of participants.

		Group		
	Chronic Pain (*n* = 20)	Acute Pain (*n* = 20)	Control (*n* = 20)	
	Mean (SD)	Mean (SD)	Mean (SD)	Significance Level
Age (years)	40.8 (1.44)	37.9 (1.32)	40.75 (1.63)	0.28
Pain duration (months)	29.1 (2.54)	1.07 (0.07)	0	*p* < 0.001
BMI	21.97 (1.54)	19.81 (0.75)	21.11 (0.81)	0.38
Height (centimeters)	167.65 (2.01)	170.5 (2.11)	171.45 (1.71)	0.36
Weight (kilograms)	61.2 (3.97)	58.1 (2.56)	60.25 (1.84)	0.75
Gender (women)	*n* = 10	*n* = 9	*n* = 10	0.98

## Data Availability

The data presented in this study are available on request from the corresponding author.
